# Pentagalloylglucose alleviates acetaminophen-induced acute liver injury by modulating inflammation via cGAS-STING pathway

**DOI:** 10.1186/s10020-024-00924-6

**Published:** 2024-09-27

**Authors:** Congyang Zheng, Yuanyuan Chen, Tingting He, Ye Xiu, Xu Dong, Xianling Wang, Xinru Wen, Chengwei Li, Qing Yao, Simin Chen, Xiaoyan Zhan, Lili Gao, Zhaofang Bai

**Affiliations:** 1 Medical School of Chinese PLA, Beijing, China; 2 Department of Gastroenterology, The Second Medical Center of Chinese PLA General Hospital, Beijing, 100853 China; 3 Department of Hepatology, The Fifth Medical Center of Chinese PLA General Hospital, Beijing, 100039 China

**Keywords:** Pentagalloylglucose, Acetaminophen, Acute liver injury, cGAS-STING pathway

## Abstract

**Background:**

The cGAS-STING pathway is an important component of the innate immune system and plays significant role in acetaminophen-induced liver injury (AILI). Pentagalloylglucose (PGG) is a natural polyphenolic compound with various beneficial effects, including anti-cancer, antioxidant, anti-inflammatory, and liver-protective properties; however, whether it can be used for the treatment of AILI and the specific mechanism remain unclear.

**Materials and methods:**

A cell culture model was created to study the effect of PGG on cGAS-STING pathway activation using various techniques including western blotting (WB), real-time quantitative polymerase chain reaction (RT-qPCR), immunofluorescence (IF), and immunoprecipitation (IP). The effect of PGG was investigated in vivo by establishing a dimethylxanthenone acetic acid (DMXAA)-mediated activation model. An AILI model was used to evaluate the hepatoprotective and therapeutic effects of PGG by detecting liver function indicators, liver histopathology, and cGAS-STING pathway-related indicators in mice with AILI.

**Results:**

PGG blocked cGAS-STING pathway activation in bone marrow-derived macrophages (BMDMs), THP-1 cells, and peripheral blood mononuclear cells (PBMCs) in vitro. Furthermore, PGG inhibited the generation of type I interferons (IFN-I) and the secretion of inflammatory factors in DMXAA-induced in vivo experiments. In addition, PGG also reduced serum levels of alanine aminotransferase (ALT), aspartate aminotransferase (AST), and alkaline phosphatase (ALP), improved liver tissue damage and apoptosis, and inhibited the cGAS-STING pathway activation caused by acetaminophen. In terms of the mechanism, PGG disrupted the connection between STING and TBK1.

**Conclusions:**

PGG exerts a protective effect against AILI by blocking the cGAS-STING pathway, offering a promising treatment strategy.

**Supplementary Information:**

The online version contains supplementary material available at 10.1186/s10020-024-00924-6.

## Introduction

Drug-induced liver injury caused by an overdose of prescription drugs and herbal or dietary supplements, can result in direct or indirect liver damage. The primary cause is often an excessive amount of acetaminophen, which can lead to acute liver failure and potentially fatal outcomes (Kumachev and Wu 2021). Notably, previous study indicated that the hepatotoxicity induced by acetaminophen overload was primarily associated with the overaccumulation of N-acetyl-p-benzoquinone imine (NAPQI), which can rapidly deplete reduced glutathione (GSH), trigger oxidative stress and mitochondrial dysfunction, causing cell death (Zhu et al. 2023). N-acetyl cysteine (NAC) is currently the only antidote approved for acetaminophen-induced liver injury (AILI); however, it is only effective in the early stages of the disease (Guo et al. 2023). Therefore, it is crucial to promptly develop new medications to treat AILI.

A recent study showed that AILI can trigger an innate immune response, which is primarily characterized by the release of damage-associated molecular patterns (DAMPs) (Roth et al. 2023), such as cytosolic DNA, from necrotic hepatocytes, which can activate the cyclic GMP-AMP synthase (cGAS)-interferon gene stimulator (STING) pathway (Jiménez-Loygorri et al. 2024). cGAS functions as a detector of cytosolic DNA, triggering STING by generating the second messenger 2′3′-cGAMP, which recruits and activates TANK-binding kinase 1 (TBK1), leading to the phosphorylation of interferon regulatory factor 3 (IRF3) and the production of type I interferon (IFN-I) (Decout et al. 2021; Sun et al. 2024). In addition, activated STING can activate the nuclear factor kappa-B (NF-κB) pathway, which up-regulates the expression of inflammatory factors, such as tumor necrosis factor-alpha (TNF-ɑ) and interleukin-6 (IL-6) (Hopfner and Hornung 2020). Interestingly, DNA produced by acetaminophen-induced hepatocyte necrosis can trigger the cGAS-STING pathway in non-parenchymal liver cells, leading to the generation of IFN-I and secretion of inflammatory factors, ultimately worsening liver damage (Yang et al. 2023; Fan et al. 2024). Consistent with these findings, recent studies have indicated that STING mutations can significantly attenuate liver injury caused by acetaminophen overload by modulating the inflammatory response (Li et al. 2023a, b, c); therefore, targeting the cGAS-STING pathway may be a promising treatment for AILI.

Pentagalloylglucose (PGG) is a polyphenolic compound with powerful antioxidant, anti-inflammatory, antibacterial, antiviral, and antitumor effects, and is used in the treatment of liver diseases (Ashibe et al. 2017; Mikolajczyk et al. 2019). It has been reported that PGG ameliorates high-fat diet-induced nonalcoholic fatty liver disease (NAFLD) and preserve the regulation of genes associated with lipid balance in mice (Kant et al. 2020). Furthermore, the combination of PGG and metformin almost completely reverses the pathophysiological changes in NAFLD in mice by inducing the expression of glycine N-methyltransferase (Yang et al. 2022). In addition, PGG exhibits hepatoprotective properties by activating Nrf2 nuclear translocation in a manner dependent on extracellular signal-regulated kinases, leading to the induction of HO-1 expression (Pae et al. 2006). PGG is crucial in the management of HCC as it triggers Huh7 cell death and improves the response to sorafenib treatment (Dong et al. 2014). However, the benefits of PGG in AILI have not yet been reported.

In the present study, we revealed that PGG significantly suppressed activation of the cGAS-STING pathway by blocking the interaction between TBK1 and STING. Notably, PGG protects against and treats AILI by suppressing the release of IFN-I, the upregulation of inflammatory factors, and the activation of the cGAS-STING pathway. In summary, these results suggest that PGG is a potential strategy for the treating patients with AILI.

## Materials and methods

### Animals

Eight-week-old C57BL/6 mice were purchased from SPF Biotechnology Co Ltd (Beijing, China) and maintained under specific-pathogen-free (SPF) conditions with a temperature of approximately 25 °C and suitable humidity. The mice were allowed free access to food and water prior to the beginning of the study.

To investigate the protective effect of PGG (HY-N0527, MCE) against AILI, we established a mouse model of AILI as described previously (Zhang et al. 2021; Yu et al. 2023). The eight-week-old male C57BL/6 mice were fasted with water for 12 h. Except for the control group, which was administered the corresponding volume of solvent, each group was injected intraperitoneally with 400 mg/kg acetaminophen (HY-66005, MCE) dissolved in warm saline, and then injected with 20 and 40 mg/kg PGG (HY-N0527, MCE) and 15 mg/kg C-176 (HY-112906, MCE) 1 h later. The mice were sacrificed after 24 h, and serum and liver tissues were collected. Liver tissues were stored partly in − 80 °C refrigerator for real-time quantitative polymerase chain reaction (RT-qPCR) experiments and partly in 4% paraformaldehyde for histological examination.

To clarify whether PGG inhibited the STING pathway in vivo in mice, we performed dimethylxanthenone acetic acid (DMXAA) induction experiments (Ding et al. 2020). Intraperitoneal injections with PGG (20 and 40 mg/kg) were performed on eight-week-old C57BL/6 mice for 1 h, followed by 25 mg/kg DMXAA (HY-10964, MCE). The mice were sacrificed 4 h later, and serum and peritoneal lavage obtained by rinsing the abdominal cavities of the mice with pre-cooled phosphate-buffered saline (PBS) were collected. Subsequently, interferon-β (IFN-β), TNF-α, and IL-6 levels were measured.

### Cells

Bone marrow-derived macrophages (BMDMs) were isolated from Female C57BL/6 mice, as previously described (Wang et al. 2020), and cultured in Dulbecco’s Modified Eagle's Medium (DMEM, PYG0074, Boster) supplemented with 10% fetal bovine serum (FBS; F101-01, Vazyme), 1% penicillin/streptomycin (P/S; ccoo4, macgene) and M-CSF (HY-P7085, MCE). The THP-1 cell line (CL-0233, Procell) and peripheral blood mononuclear cells (PBMCs) obtained from the blood of healthy volunteers were cultured in Roswell Park Memorial Institute (RPMI)-1640 medium, 10% FBS, and 1% P/S (Shi et al. 2020). HEK-293 (CL-0001, Procell) and HEK-293 T (CL-0005, Procell) cells were cultured in DMEM supplemented with 10% FBS and 1% P/S. AML12 murine hepatocytes (CL-0602, Procell) were maintained in DMEM supplemented with 10% FBS and 1% P/S.

### Cell counting kit-8 (CCK8) assay

BMDMs and THP-1 cells were seeded in 96-well plates at a concentration of 1.2 × 10^6^ cells/mL. After 12 h, cells were treated with varying concentrations of PGG for 24 h. According to the manufacturer’s instructions, a solution containing 10% CCK-8 (HY-K0301, MCE) was added to the cells and the absorbance was measured at 450 nm with an enzyme label.

### STING oligomerization

Oligomerization experiments were performed as described previously (Li et al. 2018). In brief, cell lysates were loaded in natural polyacrylamide gel electrophoresis (PAGE) gels without sodium dodecyl-sulfate (SDS), soaked for 30 min at room temperature, and then run at 80 mA for 2 h. Immunoblots were performed with the appropriate antibodies.

### Overexpression experiments

HEK-293 cells were seeded in 24-well plates at a concentration of 5 × 10^5^ cells/mL overnight and then exposed to flag-tagged plasmids (Flag-STING, Flag-IRF3, and Flag-TBK1) for 12 h. Thereafter, cells were exposed to 20 μM PGG for a duration of 6 h and samples were collected for immunoblotting and RT-qPCR.

### Immunoprecipitation (IP)

HEK-293 T cells were seeded in six-well plates at a concentration of 5 × 10^5^ cells/mL overnight and then exposed to flag-tagged plasmids (Flag-vector, Flag-TBK1, and Flag-IRF3) and ha-tagged plasmids (HA-vector, HA-STING, and HA-IRF3) for 24 h. Thereafter, cells were exposed to 20 μM PGG for a duration of 6 h. The cells were then lysed on ice, and a sample of the liquid above the sediment was collected as the input. The remaining supernatant was exposed to Anti-FLAG®M2Aftix gel (A2220, MilporeSigma) for 4 h, followed by four rounds of washing with the lysate. Finally, the samples were subjected to immunoblotting (Li et al. 2016).

### Immunofluorescence (IF)

BMDMs and THP-1 cells were treated with 4% paraformaldehyde at 37 °C for 20 min. They were then permeabilized with 2% TritonX-100 for 10 min at room temperature and sealed with rapid closure solution for 1 h. Next, the cells were treated with the primary antibody for 12 h and incubated for 1 h with the Goat Anti-rabbit IgG-Alexa Fluor 594 (1:500; P03S16S, gene-protein Link) or 488 (1:500; P03S14S, gene-protein Link). Nuclei were stained using Hoechst 33258 staining solution (E607301-0005, Sangong Biotech). The following antibodies were used: rabbit anti-STING (1:100; 80165-1-RR, Proteintech), mouse anti-GM130 (1:500; 66662 1-Ig, Proteintech), rabbit anti-IRF3 (1:200; 11904S, CST), rabbit anti-P65 (1:100; 8242S, CST). Data analysis was performed using ImageJ software.

### Western bloting

According to the previous method, the protein samples were separated using 10% sodium dodecyl sulfate gel electrophoresis (SDS-PAGE) and transferred to PVDF membranes that were incubated with the corresponding primary antibodies for 12 h at 4 °C, followed by 1 h of Corresponding secondary antibodies at room temperature (Wang et al. 2019). Analyses were performed using a chemiluminescence kit (YA0372-1pk, Solarbio). In this study, we used p-IRF3 (GTX86691, GeneTex), p-IRF3 (ab76493, Abcam), p-STING (19851-1-AP, Proteintech), IRF3 (ab68481, Abcam) and HSP90 (13171-1-AP, Proteintech).

### RNA extraction and real-time quantitative PCR

The RT-qPCR reactions were quantified in real time using Taq Pro Universal SYBR qPCR Master Mix (Q712-02, Vazyme) by QuantStudio™ 6 Flex Detection System. Total RNA was isolated from animal tissues and cells in the experiment by utilizing the Trizol method. cDNA was generated with the HiScript II Q RT SuperMix (R223-01, Vazyme) following RNA quantification as per the manufacturer's instructions. The primer sequences are shown in Fig. S3, and the relative gene expression levels were calculated using the 2^−∆∆Ct^ method with ACTIN as an internal reference.

### Liver biochemical and oxidative stress analysis

Serum alanine aminotransferase (ALT; C009-2-1, Nanjing Jiancheng) and aspartate aminotransferase (AST; C010-2-1, Nanjing Jiancheng) levels and alkaline phosphatase (ALP; C059-2-2, Nanjing Jiancheng) were measured following the manufacturer's instructions to assess the levels of liver injury. Mouse liver tissues were processed according to the reagent vendor's protocol and supernatants were collected for the detection of malondialdehyde (MDA; E-BC-K025-M, Elabscience), reduced glutathione (GSH; E-BC-K030-M, Elabscience) and superoxide dismutase (SOD; E-BC-K020-M, Elabscience) to assess the levels of oxidative stress damage.

### Enzyme-linked immunosorbent assay (ELISA)

IFN-β, IL-6, and TNF-α levels in mouse serum and peritoneal lavage fluid were measured according to the manufacturer’s instructions (E607301-0005; 1210602; 1217202, DAKEWE). Levels of 2′3′-cGAMP in cell lysates and murine liver tissues were using the 2′3′-cGAMP Competitive ELISA Kit (501700, Cayman Chemical).

### Histological examination and immunohistochemistry (IHC)

The 4% paraformaldehyde-fixed mouse liver tissues were dehydrated and embedded in paraffin. Liver sections were stained with hematoxylin and eosin (HE), terminal deoxynucleotidyltransferase-mediated dUTP-biotin nick end labeling (TUNEL), or prepared for immunohistochemical staining. For immunochemistry staining, the sections were stained with an anti-STING (19851-1-AP, Proteintech).

### Detection of mtDNA in cytosolic extracts

AML 12 cells were seeded in 24-well plates at a density of 5 × 10^5^ cells/mL and exposed to acetaminophen (10 mM) for 24 h with or without PGG (20 μM). Cells were lysed in fractionation buffer (150 M NaCl, 15 mM HEPES, 1 × Protease Inhibitor, 20 µg/mL digitonin, DEPC water) for 10 min at 4 °C. Cell lysates were centrifuged thrice at 1000 × *g* for 3 min, and the supernatant was transferred to a fresh tube and spun at 17,000 *g* for 10 min to obtain the cytoplasmic fraction. DNA was isolated from the cytosolic fraction using a Qiagen DNeasy blood & tissue kit (69504, QIAGEN). The nuclear fraction served as a normalization control, and mtDNA gene (ND1) expression was determined using RT-qPCR.

### Statistical analysis

Data analysis was performed using the Prism 8 software. Multiple group comparisons were analyzed using unpaired one-way analysis of variance (ANOVA), and two group comparisons were analyzed using the unpaired Student’s t-test to calculate statistical differences. The experimental results of this study were expressed as mean ± standard deviation (SD). Statistical significance was set at p < 0.05.

## Results

### PGG inhibits cGAS-STING pathway activation in BMDMs

**Fig. 1:**
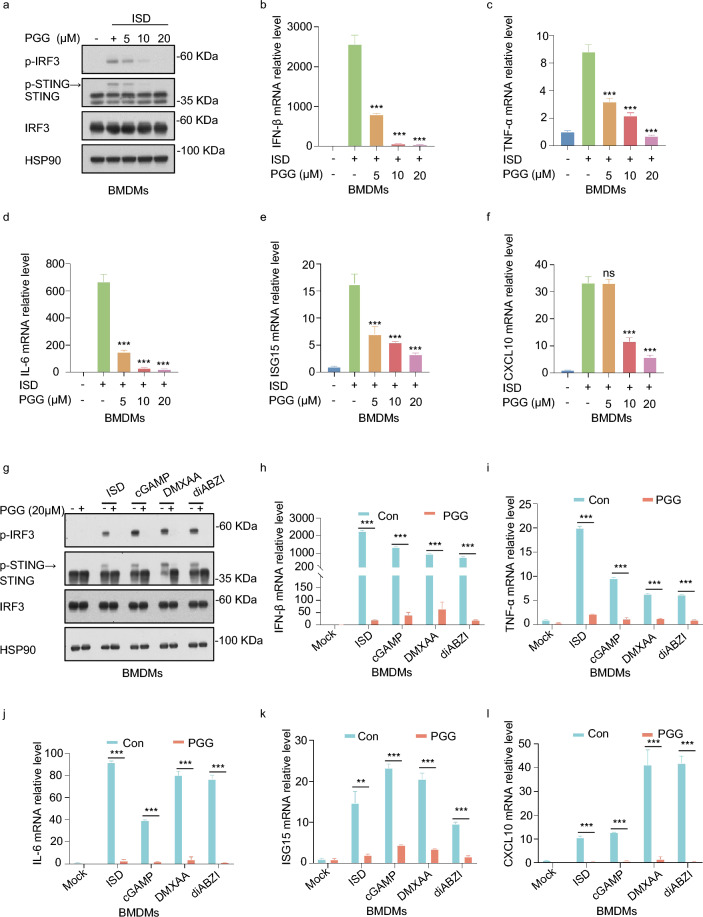
PGG inhibits cGAS-STING pathway activation in BMDMs. **a** BMDMs were pre-exposed with or without PGG (5, 10, 20 µM) 1 h before being activated by ISD for 2 h. Immunoblot analysis was performed to assess the levels of p-IRF3, IRF3, and STING. **b**–**f** BMDMs were pre-exposed to either DMSO or PGG (5, 10, 20 µM) for 1 h before being activated with ISD for 4 h. The RT-qPCR analysis revealed the levels of IFN-β (**b**), TNF-α (**c**), IL-6 (**d**), ISG15 (**e**) and CXCL10 (**f**). **g** BMDMs were pretreated with or without PGG (5, 10, 20 µM) for 1 h and then stimulated with ISD, 2′3′-cGAMP, diABZI, or DMXAA for 2 h. Cell lysates were collected to detect the expression of relevant proteins. **h**–**l** RT-qPCR assays for IFN-β (**h**), TNF-α (**i**), IL-6 (**j**), ISG15 (**k**) and CXCL10 (**l**) in BMDMs. The data were presented as means ± standard deviation (SD). **P < 0.01, ***P < 0.001. *ns* not significant

Before exploring the role of PGG (Fig. S1) in the cGAS-STING pathway, we first assessed the cytotoxicity of PGG in BMDMs, and the results suggested that PGG was non-toxic at < 60 µM (P > 0.05) (Fig. S2a). Next, BMDMs were pretreated with PGG (5, 10, or 20 µM) for 1 h and then stimulated with the cGAS agonist interferon stimulatory DNA (ISD) to model cGAS-STING activation. WB results showed that the PGG gradient inhibited the phosphorylation of IRF3 and STING compared to the ISD group (Fig. 1a). Activation of the cGAS-STING pathway induces downstream transcriptional expression of IFN-I, interferon stimulatory gene 15 (ISG15), and C-X-C motif chemokine ligand 10 (CXCL10), which initiate innate immune responses (Chen and Xu 2023). In addition, STING activates the NF-κB pathway and induces the expression of genes such as TNF-α and IL-6 (Bakhoum et al. 2018). Therefore, we examined the effect of PGG pretreatment on the expression of pathway-associated genes and determined that PGG inhibited the mRNA expression of IFN-β, TNF-α, IL-6, ISG15, and CXCL10 in ISD-treated BMDMs (Fig. 1b–f). To investigate more deeply the effect of PGG on cGAS-STING pathway activation, 2′3'-cGAMP, DMXAA and diABZI that are all STING agonists, were added to induce pathway activation. Next, we analyzed the expression of cGAS-STING pathway-related proteins and genes in parallel. The results suggested that PGG was able to inhibit the phosphorylation of IRF3 and STING (Fig. 1g) and reduce the levels of IFN-β, TNF-α, IL-6, ISG15, and CXCL10 (Fig. 1h–l). Collectively, these results indicate that PGG can widely inhibit the activation of the cGAS-STING pathway in BMDMs.

### PGG suppresses the release of IFN-I and inflammatory factors due to cGAS-STING pathway activation in THP-1 and PBMCs

**Fig. 2:**
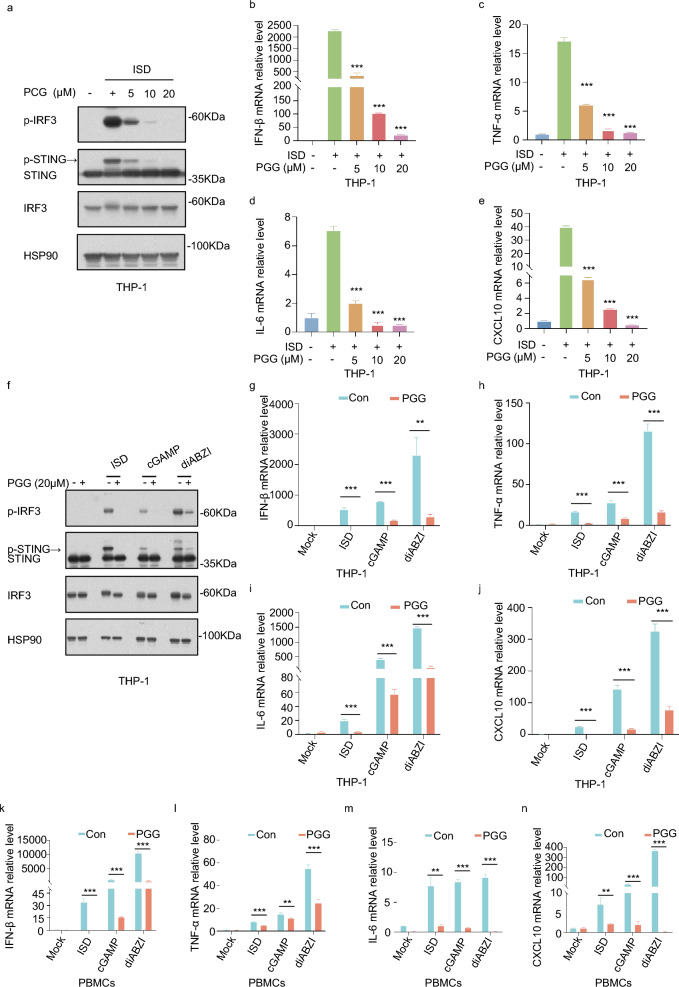
In THP-1 and PBMCs, PGG inhibits the activation of the cGAS-STING pathway. **a** PMA-primed THP-1 were pre-exposed with or without PGG (5, 10, 20 µM) for 1 h before being activated by ISD for 2 h. Immunoblotting was used to detect p-IRF3, IRF3, and STING. **b**–**e** PMA-primed THP-1 were pre-exposed with DMSO or PGG (5, 10, 20 µM) for 1 h before being activated with ISD for 4 h. The mRNA levels of IFN-β (**b**), TNF-α (**c**), IL-6 (**d**), and CXCL10 (**e**) were detected by RT-qPCR. **f** Western blot assays of p-IRF3, IRF3, and STING in PMA-primed THP-1. **g**–**j** RT-qPCR assays for IFN-β (**g**), TNF-α (**h**), IL-6 (**i**), and CXCL10 (**j**) in PMA-primed THP-1. **k**–**n** RT-qPCR was utilized to measure the expression levels of the IFN-β (**k**), TNF-α (**l**), IL-6 (**m**), and CXCL10 (**n**) genes in PBMCs. The data were presented as means ± SD. **P < 0.01, ***P < 0.001

Next, we explore the effect of PGG on the activation of the cGAS-STING pathway in human cells. Similarly, CCK8 assay showed that PGG on THP-1 cells was essentially non-toxic at < 60 µM (Fig. S2b). We treated PMA-induced differentiated THP-1 cells with PGG (0, 10, or 20 µM) for 1 h followed by ISD treatment. Compared to the ISD group, PGG treatment reduced the expression of p-IRF3 and p-STING protein in the THP-1 cells (Fig. 2a). In addition, we examined the expression of IFN-β, TNF-α, IL-6 and CXCL10 using RT-qPCR and determined that PGG can reduce their expression (Fig. 2b–e).

To further investigate the effect of PGG on the activation of cGAS-STING pathway, 2′3′-cGAMP, diABZI was used to treat PMA-induced differentiated THP-1 cells. The WB results showed that, consistent with the results of ISD treatment, PGG inhibited IRF3 and STING phosphorylation (Fig. 2f). The RT-qPCR experiments showed that PGG inhibited the expression of IFN-β, TNF-α, IL-6 and CXCL10 in THP-1 cells (Fig. 2g–j). Similarly, we used ISD, 2′3′-cGAMP and diABZI to treat PBMCs, and investigated the effect of PGG on the activation of the cGAS-STING pathway. The RT-qPCR results showed that compared with the blank group, IFN-β, TNF-α, IL-6, and CXCL10 levels in the model groups significantly elevated, however, PGG administration reduced the increased levels (Fig. 2k–n). These studies showed that PGG markedly inhibit cGAS-STING pathway activation in THP-1 cells and PBMCs.

### PGG suppresses cGAS-STING pathway activation by inhibiting the interaction between STING and TBK1

**Fig. 3:**
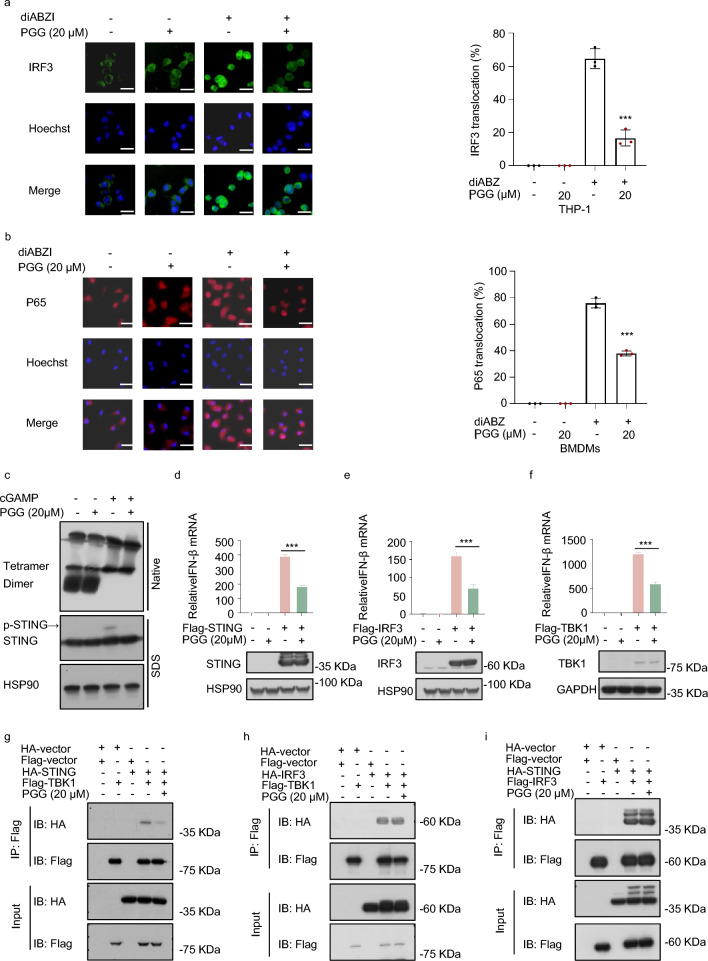
PGG suppresses cGAS-STING pathway activation via inhibiting the interaction between STING and TBK1. **a**, **b** PMA-primed THP-1 or BMDMs were treated with DMSO or PGG at a concentration of 20 µM for 1 h, followed by stimulation with diABZI for 2 h. Immunostaining of IRF3 (**a**) and P65 (**b**) (n = 3). Scale bar, 10 μm. **c** After PGG at a concentration of 20 µM pretreatment for 1 h, BMDMs were stimulated with 2′3′-cGAMP for half an hour, followed by analysis of STING oligomerization and phosphorylation using immunoblotting. **d**–**f** Flag-tagged plasmid (Flag-STING, Flag-IRF3, Flag-TBK1) were transfected into HEK-293 cells for 12 h. Afterwards, cells were exposed to 20 μM PGG for a duration of 6 h and then subjected to WB and RT-qPCR. **g**–**i** Flag-tagged plasmid (Flag-vector, Flag-TBK1, Flag-IRF3) and ha-tagged plasmid (HA-vector, HA-STING, HA-IRF3) were transfected into HEK-293 T cells for 24 h. Afterwards, cells were exposed to 20 μM PGG for a duration of 6 h and then subjected to immunoprecipitation using Anti-FLAG®M2Aftix gel for a period of 4 h in preparation for immunoblotting with the specified antibodies. The data were presented as means ± SD. ***P < 0.001

cGAS induced oligomerization of STING by synthesizing 2′3′-cGAMP, leading to its movement from the endoplasmic reticulum to the Golgi apparatus (Gaidt et al. 2017), which then recruited TBK1 and phosphorylates IRF3, inducing the release of IFN-I and simultaneously activated STING can also activate the NF-κB pathway, causing P65 to enter the nucleus and triggering the generation of inflammatory factors (Zhang et al. 2019; Li et al. 2024). Next, we examined the effect of PGG on ISD-stimulated 2′3'-cGAMP synthesis, and the results showed a decrease in 2′3'-cGAMP content after PGG treatment (Fig. S4). Furthermore, we evaluated the effects of PGG on the nuclear translocation of P65 and IRF3. The results revealed that PGG inhibited the nuclear entry of P65 in BMDMs, and IRF3 in THP-1 cells, and the quantitative results were consistent with our observations (Fig. 3a, b). Next, we evaluated the effect of PGG on STING oligomerization, and the results showed that PGG did not affect STING oligomerization; therefore, we hypothesized that it affects other processes of STING or downstream signaling to play a role (Fig. 3c). Next, we investigated whether PGG affects the translocation of STING to the Golgi. Confocal microscopy images showed that STING was transported to the Golgi and colocalized with the Golgi after 2′3′-cGAMP stimulation in THP-1 cells. However, PGG did not affect the colocalization of STING and Golgi (Fig. S5). To identify the protein targets of PGG, we overexpressed the Flag tagged plasmids (STING, IRF3, and TBK1) in 293 cells to measure IFN-β mRNA expression, and determined that PGG significantly reduced the mRNA expression levels of IFN-β induced by STING, IRF3, and TBK1, suggesting that the action of PGG may be related to STING (Fig. 3d–f). Based on the above experimental results, we investigated the effects of PGG on the interactions between TBK1 and STING, IRF3 and STING, and TBK1 and IRF3. Our findings showed that PGG blocked the interaction between TBK1 and STING, consequently influencing the activation of the cGAS-STING pathway (Fig. 3g–i). These results suggested that PGG is a broad-spectrum inhibitor of the cGAS-STING pathway.

### PGG inhibits the activation of STING and inflammatory response in vivo

**Fig. 4:**
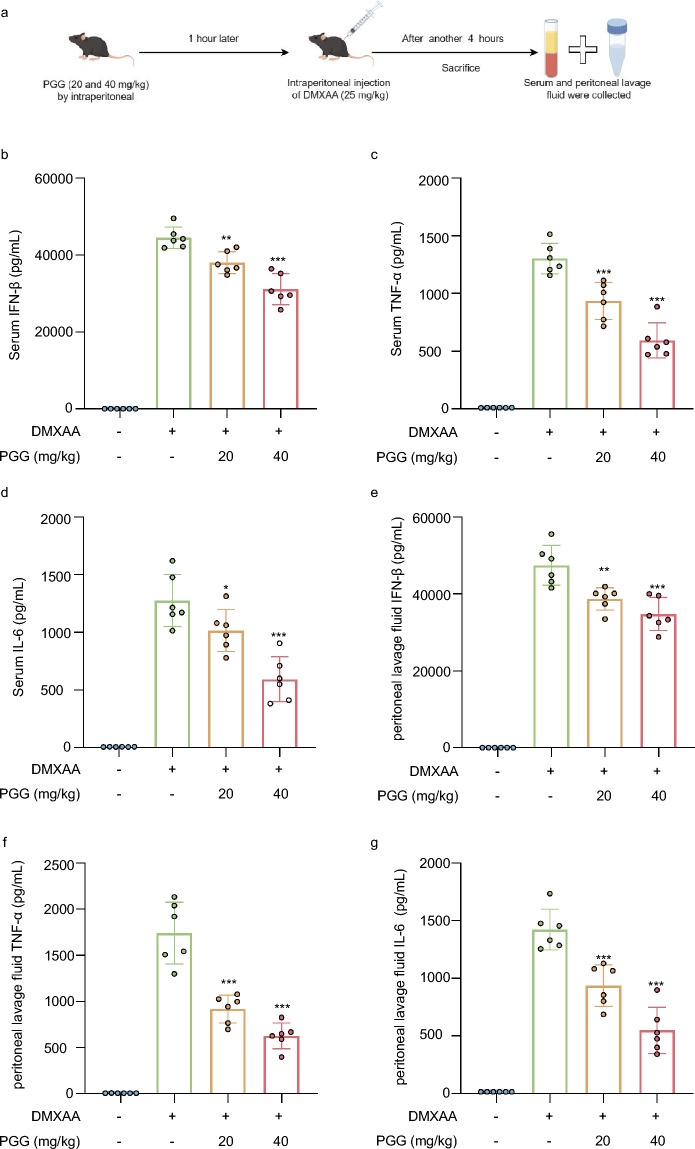
PGG inhibits the activation of STING and inflammatory response in vivo. **a** Schematic representation of an experiment performed on mice with DMXAA to induce STING activation. **b**–**d** Mouse serum was collected to measure the levels of IFN-β (**b**), TNF-α (**c**) and IL-6 (**d**) using ELISA kits (n = 6). **e**–**g** Mouse peritoneal lavage fluid was collected to measure the levels of IFN-β (**e**), TNF-α (**f**) and IL-6 (**g**) by ELISA kits (n = 6). The data were presented as means ± SD. *P < 0.05, **P < 0.01, ***P < 0.001 vs. the model group

Subsequently, we assessed the effects of PGG on STING pathway activation in vivo. DMXAA, a murine agonist of STING, can induce the release of IFN-I and mediate the upregulation of the NF-κB pathway that leads to increased secretion of inflammatory factors such as TNF-ɑ and IL-6 by activating STING. Therefore, we established a DMXAA agonist model in which mice were injected intraperitoneally with different doses of PGG (20 and 40 mg/kg) for 1 h, followed by DMXAA (25 mg/kg) injection. Serum and intraperitoneal lavage fluid were collected 4 h later (Fig. 4a). Next, we measured the expression of IFN-β, TNF-α, and IL-6 in serum and peritoneal lavage fluid using ELISA kits. The results showed that the expression IFN-β, TNF-α, and IL-6 in the serum and peritoneal lavage fluid was decreased after administration of PGG compared to the model group, which showed that PGG inhibited the activation of STING and related-inflammatory response (Fig. 4b–g).

### PGG protect acetaminophen- induced acute liver injury

**Fig. 5:**
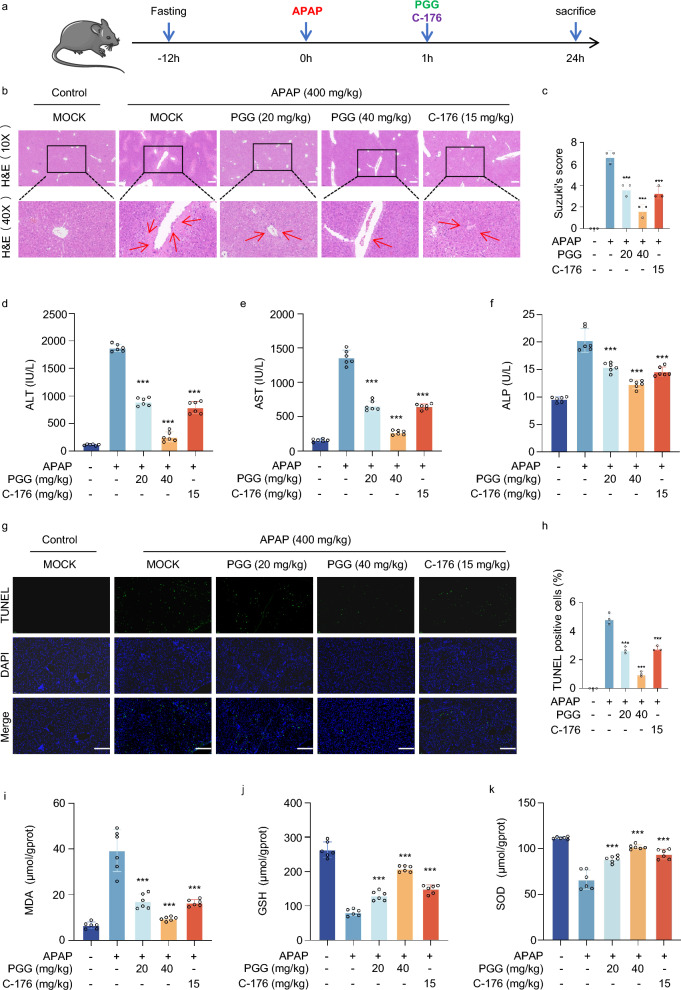
PGG protect acetaminophen-induced acute liver injury. **a** Schematic illustration representing an experiment conducted on mice to induce acute liver injury using acetaminophen. **b** Representative H&E staining of liver tissues from AILI mice (n = 3). Scale bar: 100 µm. The red arrow points to the area of the liver lesion. (c) Grading the histological severity of liver injury induced by acetaminophen using the Suzuki score (n = 3). **d**–**f** Serum levels of ALT, AST and ALP from AILI mice (n = 6). **g**, **h** Representative images and quantification of TUNEL assay of liver tissues from AILI mice (n = 3). Scale bar: 100 µm. **i**–**k** Hepatic tissue MDA, GSH and SOD levels were detected by biochemical kits (n = 6). The data were presented as means ± SD. ***P < 0.001 vs. the model group

To determine whether PGG protects against acetaminophen-induced hepatocytotoxicity, we developed an AILI model (Fig. 5a). Recent studies have shown that the cGAS-STING pathway is involved in AILI, and that inhibition of this pathway can play a protective role against liver injury. Furthermore, C-176, an inhibitor of STING, has been previously reported to alleviate liver inflammation and injury through the STING pathway (Chen et al. 2022). To verify that PGG inhibits AILI through the STING pathway, we used C-176 as a positive control. Histological changes in the liver were assessed 24 h after acetaminophen stimulation, and balloon degeneration and necrosis were significantly reduced after the administration of PGG or C-176 (Fig. 5b, c). In addition, consistent with the histological results, PGG or C-176 treatment significantly reduced the alanine aminotransferase (ALT), aspartate aminotransferase (AST), and alkaline phosphatase (ALP) levels in mice with AILI (Fig. 5d-f). Acetaminophen overload can cause cell necrosis and apoptosis through oxidative damage, which is associated with liver injury. The results of TUNEL staining showed that hepatocyte apoptosis was reduced by PGG or C-176 compared to that in the acetaminophen group (Fig. 5g, h). Moreover, MDA is a marker of lipid peroxidation in the liver tissue, and oxidative stress is strongly associated with acetaminophen-induced hepatotoxicity due to acetaminophen (Guo et al. 2019). As shown in Fig. 5i-k, administration of PGG or C-176 reduced MDA levels and increased the vitality of SOD and GSH in the liver tissue induced by acetaminophen, thus exerting a protective role against AILI by inhibiting oxidative stress. Collectively, these data suggested that PGG effectively reduced hepatic histopathological injury, abnormal liver function, hepatic apoptosis and oxidative damage in acetaminophen-treated mice.

### PGG suppresses cGAS-STING pathway activation in mice with AILI

It is commonly acknowledged that acetaminophen overload can cause oxidative stress, leading to mitochondrial damage and cell death and resulting in the release of mtDNA into the cytoplasm, which can activate the cGAS-STING pathway and cause secondary liver damage (Dong et al. 2024). To confirm this mechanism, we performed nuclear and cytoplasmic isolation and detected mtDNA levels using RT-qPCR. The mtDNA levels were elevated in the cytoplasmic fraction of acetaminophen-treated AML 12 cells compared to those in the control, whereas the administration of PGG did not alter the mtDNA levels (Fig. S6).

**Fig. 6:**
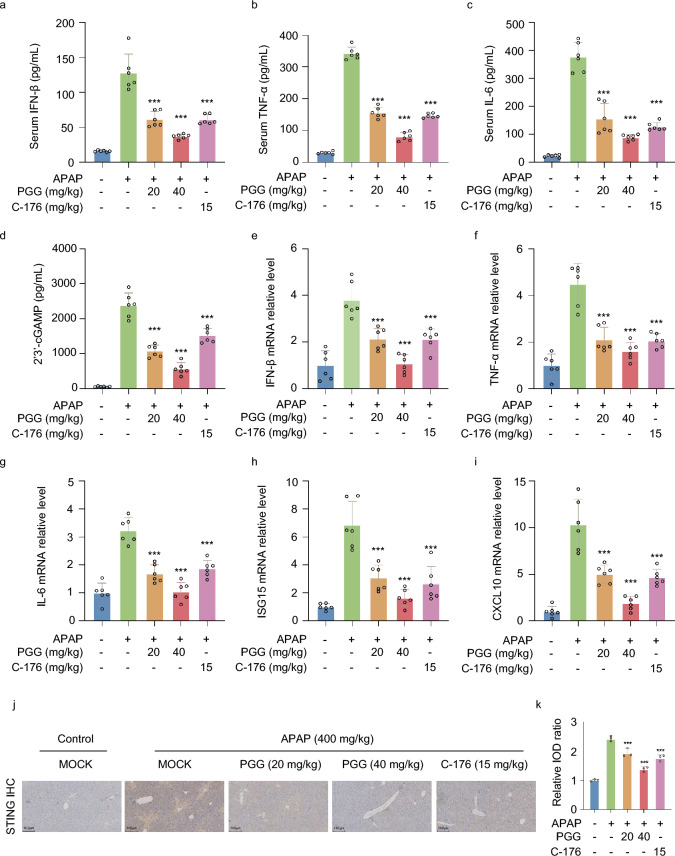
PGG suppresses cGAS-STING Pathway activation in AILI mice. **a**–**c** The levels of IFN-β, TNF-α and IL-6 from AILI mice serum were detected by ELISA kits (n = 6). **d** The levels of 2′3′-cGAMP from AILI mice liver tissues were detected by ELISA kits (n = 6). **e**–**i** The mRNA levels of cGAS-STING pathway-related genes (IFN-β, TNF-α, IL-6, ISG15 and CXCL10) were measured by RT-qPCR assays in liver tissues from AILI mice (n = 6). **j**, **k** Representative images and quantification of STING expression in liver tissues (n = 3). Scale bar: 100 µm. The data were presented as means ± SD. ***P < 0.001 vs. the model group

To determine whether PGG can inhibit acetaminophen-induced hepatic inflammatory and cGAS-STING pathway activation to liver injury, we examined the levels of cGAS-STING pathway-driven IFN-β and inflammation factors in the AILI model. The results showed that serum IFN-β, TNF-α, and IL-6 levels were significantly higher in the acetaminophen group compared with those in the control group, and that PGG and C-176 significantly reduced serum IFN-β, TNF-α, and IL-6 levels (Fig. 6a–c).

To investigate the protective mechanism of PGG against AILI, we assessed 2′3′-cGAMP levels and mRNA expression of cGAS-STING pathway-related genes in an AILI mouse model. The ELISA results showed that acetaminophen treatment significantly increased 2′3′-cGAMP levels in mouse liver tissues, whereas PGG and C-176 inhibited 2′3′-cGAMP production (Fig. 6d). Furthermore, in comparison to the acetaminophen group, IFN-β, TNF-α, IL-6, ISG15, and CXCL10 mRNA levels were significantly reduced in the PGG or C-176 groups (Fig. 6e–i). IHC staining also confirmed the promotional effect of acetaminophen on STING expression in mouse liver, which was effectively reversed by PGG and C-176 treatments (Fig. 6j, k). In conclusion, these findings indicate that PGG exerts a protective effect against AILI by inhibiting the activation of the cGAS-STING pathway in an AILI model.

## Discussion

Excessive acetaminophen intake leading to acute liver damage is a prevalent public health issue worldwide and requires resolution (Stravitz and Lee 2019). As our current understanding of the disease is lacking, treatments are primarily focused on the early effective stage of the disease. However, the inflammatory response induced by acetaminophen can amplify liver injury, which is why a large amount of hepatocellular necrosis can occur even if the initiating factors are removed (Wang et al. 2022). AILI has two distinct phases: the initial stage involves damage from acetaminophen and its harmful by-products, whereas the subsequent stage is characterized by an innate immune reaction triggered by cell necrosis (Widjaja et al. 2021). A growing number of studies have suggested that counteracting the activation of the innate immunity may be an effective means of treating AILI. Acetaminophen overload later causes hepatocyte death and the release of a large amount of cytosolic DNA, which can be sensed by cGAS. This leads to the activation of STING, thereby inducing IFN-I production and triggering the release of NF-κB pathway-related inflammatory factors, which causes massive hepatocyte necrosis (Dong et al. 2023). Therefore, inhibition of cGAS-STING pathway activation can alleviate AILI.

Current drugs for the treatment of AILI have limited clinical use owing to their narrow time windows (Ouyang et al. 2024). Therefore, drugs that can be used to treat massive necrosis of hepatocytes caused by an excessive inflammatory response due to acetaminophen overdose require exploration. Natural drugs are considered a good choice for AILI because of their low cost, low toxicity, and high bioavailability (Li et al. 2023a, b, c). PGG has excellent anti-inflammatory and antioxidant effects, and is used in the treatment of liver diseases; however, its use in the treatment of AILI has not been reported.

In the present study, PGG significantly inhibited the activation of the cGAS-STING pathway induced by ISD, 2′3′-cGAMP, DMXAA, and diABZI, and the same effect was observed in human THP-1 cells and PBMCs, suggesting that PGG is a broad-spectrum inhibitor of the cGAS-STING pathway. Further exploration of the underlying mechanism revealed that PGG suppressed the interaction between STING and TBK1. In conjunction with the in vivo experiment, we determined that PGG can inhibit IFN-I production and the inflammatory response in the DMXXAA agonist model. Furthermore, PGG treatment ameliorated liver tissue injury and hepatocyte apoptosis, reduced serum liver function indices, decreased the production of inflammatory factors, and lowered the activation of the cGAS-STING pathway, suggesting that it indeed exerted a protective effect against AILI. In conclusion, these findings confirm that PGG suppresses the immune response by affecting the cGAS-STING pathway induced by acetaminophen overload. C-176, a selective and potent STING inhibitor, covalently binds to C91 to significantly reduce serum IFN-I levels and inflammatory factor release (Wu et al. 2022; Li et al. 2023a, b, c). In our study, administration of C-176 reduced serum ALT, AST, and ALP levels and inhibited IFN-β, TNF-α, IL-6, ISG15, and CXCL10 gene expression, thereby alleviating AILI. It has been reported in the literature that pre-addition of RU.521, a selective cGAS inhibitor, significantly reduced the ALT and AST levels and inhibited the expression of IFN-β genes in acetaminophen-treated mice, and improved the survival rate of mice (Wang et al. 2023). Therefore, targeting cGAS-STING may be an option for AILI treatment.

Notably, we only evaluated PGG treatment for protection against AILI and did not test the long-term effects of PGG, which requires further research. In addition, PGG can also protect hepatocytes from oxidative stress and inhibit the NF-κB pathway to exhibit a good anti-inflammatory effect (Lee et al. 2011; Tong et al. 2021), which also suggests that PGG may also play a protective role against AILI through other targets and mechanisms. Therefore, the use of PGG to treat liver injury caused by other drugs requires further investigation.

## Conclusion

**Fig. 7:**
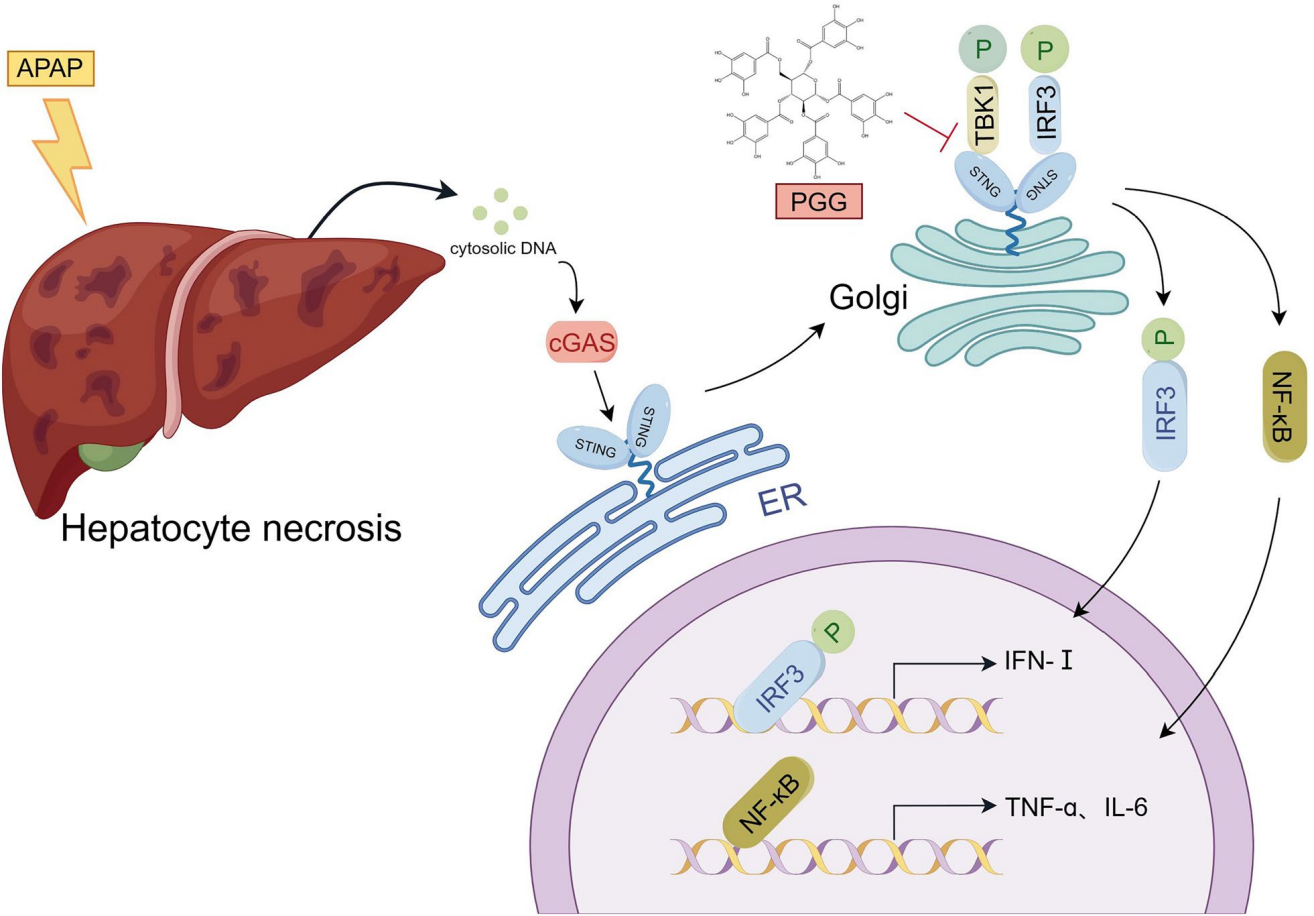
Graphic abstract. A schematic diagram showing that PGG attenuates AILI via inhibiting cGAS-STING pathway, involving suppressing binding between STING and TBK1

In summary, PGG, a natural polyphenol, significantly inhibited the activation of the cGAS-STING pathway by suppressing the binding between STING and TBK1 in vitro. In addition, it alleviated IFN-I release and inflammatory reactions in DMXAA-treated mice. Furthermore, PGG notably attenuated AILI in mice by inhibiting the inflammatory response associated with the activation of the cGAS-STING pathway. The present study revealed a novel mechanism through which PGG protects against AILI, offering a new approach for treating this condition (Fig. 7).

## Supplementary Information

Additional file 1.

## Data Availability

Data are available upon reasonable request from the corresponding author.

## Abbreviations

PGG: Pentagalloylglucose
AILI: Acetaminophen-induced liver injury
ALT: Alanine aminotransferase
AST: Aspartate aminotransferase
ALP: Alkaline phosphatase
BMDMs: Bone marrow-derived macrophages
PBMCs: Peripheral blood mononuclear cells
NAPQI: N-acetyl-p-benzoquinone imine
NAC: N-acetyl cysteine
DAMPs: Damage-associated molecular patterns
cGAS: Cyclic GMP-AMP synthase
STING: Interferon gene stimulator
TBK1: TANK-binding kinase 1
IRF3: Interferon regulatory factor 3
NF-κB: Nuclear factor kappa-B
IFN-I: Type 1 interferon
IFN-β: Interferon-β
TNF-ɑ: Necrosis factor-alpha
IL-6: Interleukin- 6
NAFLD: Nonalcoholic fatty liver disease
CCK8: Cell counting kit-8 assay
ELISA: Enzyme-linked immunosorbent assay
IF: Immunofluorescence
IP: Immunoprecipitation
IHC: Immunohistochemistry
HE: Hematoxylin and eosin
TUNEL: Terminal deoxynucleotidyltransferase-mediated dUTP-biotin nick end labeling
RT-qPCR: Real-time quantitative PCR
ISD: Interferon stimulatory DNA
ISG15: Interferon stimulatory gene 15
CXCL10: C-X-C motif chemokine ligand 10
MDA: Malondialdehyde
GSH: Reduced glutathione
ANOVA: Analysis of variance
SD: Standard deviation
SOD: Superoxide dismutase
