# Supplementary material for: Pentagalloylglucose alleviates acetaminophen-induced acute liver injury by modulating inflammation via cGAS-STING pathway
Source: Mol Med. 2024 Sep 27;30:160. doi: 10.1186/s10020-024-00924-6 (PMC11428449; doi:10.1186/s10020-024-00924-6)
Supplement: Supplementary file 1 — Additional file 1. [file 10020_2024_924_MOESM1_ESM.pdf]

Fig. S1 The Chemical structure of PGG

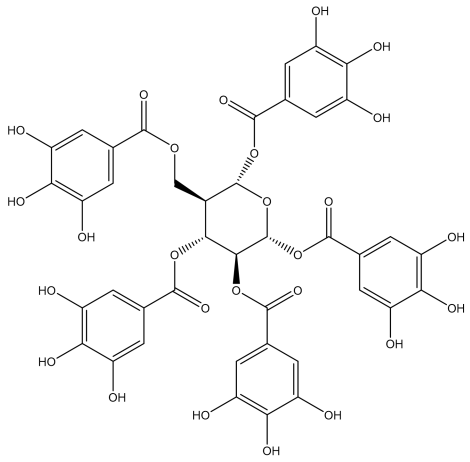

Fig. S2 Cell viability assays of PGG in BMDMs and THP-1

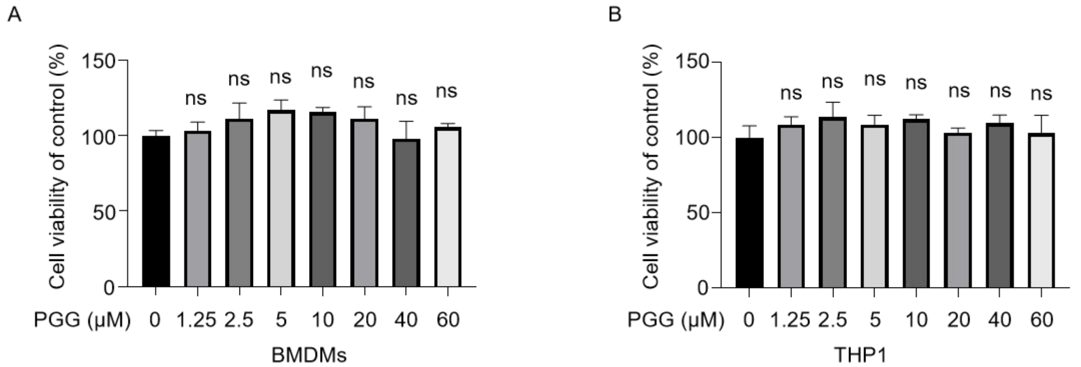


(a) The effect of different concentrations of PGG on BMDMs viability was determined by using the CCK8 assay.

(b) The effect of different concentrations of PGG on PMA-primed THP-1 viability was determined by using the CCK8 assay.

Fig. S3 Primers for RT-qPCR used in this study

| Gene Name | Forward primer (5′ → 3′) | Reverse primer (5′ → 3′) |
|---|---|---|
| Human IFN-β | TCCAAATTGCTCTCCTGTTG | GCAGTATTCAAGCCTCCCAT |
| Human IL-6 | ACTCACCTCTTCAGAACGAATTG | CCATCTTTGGAAGGTTCAGGTTG |
| Human TNF-α | CCTCTCTCTAATCAGCCCTCTG | GAGGACCTGGGAGTAGATGAG |
| Human CXCL10 | TGGCATTCAAGGAGTACCTC | TTGTAGCAATGATCTCAACACG |
| Mouse IFN-β | TCCGAGCAGAGATCTTCAGGAA | TGCAAC CACCACTCATTCTGAG |
| Mouse IL-6 | CACTTCACAAGTCGGAGGCT | CTGCAAGTGCATCATCGTTGT |
| Mouse TNF-α | CAGGCGGTGCCTATGTCT | CGATCACCCCGAAGTTCAGTAG |
| Mouse ISG15 | GGTGTCCGTGACTAACTCCAT | CTGTACCACTAGCATCACTGTG |
| Mouse CXCL10 | ATCATCCCTGCGAGCCTATCCT | GACCTTTTTTGGCTAAACGCTTTC |
| Human actin | CATGTACGTTGCTATCCAGGC | CTCCTTAATGTCACGCACGAT |
| Mouse actin | GGCTGTATTCCCCTCCATCG | CCAGTTGGTAACAATGCCATGT |
| ND1 | CGGGCTACTACAACCCTTCG | GCGATGGTGAGAGCTAAGGT |
| 18S | TAGAGGGACAAGTGGCGTTC | CGCTGAGCCAGTCAGTGT |

Fig. S4 PGG reduces the synthesis of 2′-3′-cGAMP

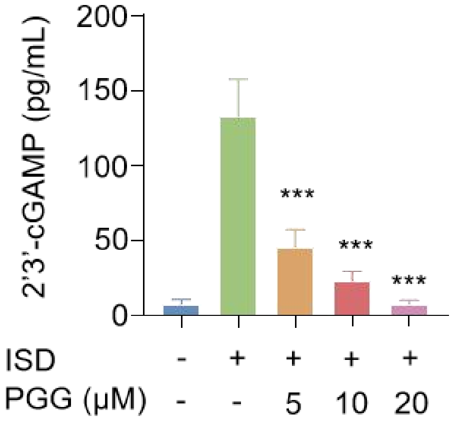


（a）BMDMs were first treated with DMSO or PGG (5, 10, 20 μM) for 1 h and then stimulated with ISD for 2 h. Whole cell lysates were collected and assayed for 2′-3′-cGAMP by ELISA. The data were presented as means ± standard deviation (SD). ***P < 0. 001.

Fig. S5 The effect of PGG on STING and Golgi co-localization

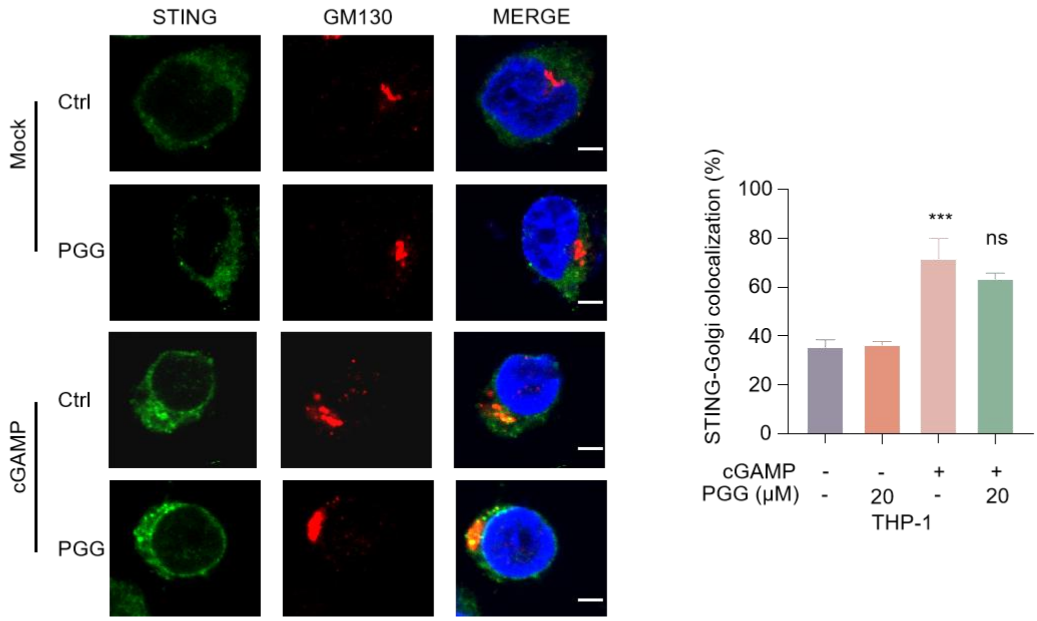


（a）Confocal microscopy of PMA-primed THP-1 cells were treated with DMSO or PGG at a concentration of 20 μM for 1 h, followed by stimulation with 2′-3′-cGAMP for 2 h. Colocalization of the Golgi apparatus (GM130, red) and STING (green) in cells was analyzed by ImageJ (right). The data were presented as means ± SD. ***P < 0. 001 vs. the control group. NS: not significant vs. the model group.

Fig. S6 Acetaminophen induces mtDNA release into cytoplasm

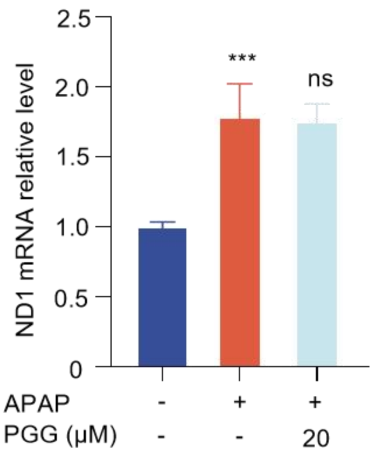


（a）AML 12 cells were treated by APAP (10 mM) with or without PGG (20 μM) for 24 h and mtDNA content in cells was determined by RT-qPCR. The data were presented as means ± SD. ***P < 0. 001 vs. the control group. NS: not significant vs. the model group.
